# Supplementary material for: Albumin in patients with liver disease shows an altered conformation
Source: Commun Biol. 2021 Jun 14;4:731. doi: 10.1038/s42003-021-02269-w (PMC8203801; doi:10.1038/s42003-021-02269-w)
Supplement: Supplementary file 2 — Supplemental Material [file 42003_2021_2269_MOESM2_ESM.pdf]

SUPPLEMENTARY MATERIALS

Supplementary Table 1: Plasma variables related to liver disease.

Pat.	Bilirubin	Albumin	Creatinine	AST	ALT	GGT	AP	INR
	**mg/dL**	**g/dL**	**mg/dL**	**U/L**	**U/L**	**U/L**	**U/L**	
P1	34.3	3.0	0.88	225	112	49	67	2.56
P2	5.0	2.8	2.34	38	17	32	102	1.63
P3	2.4	5.0	0.78	29	19	208	131	1.42
P4	1.1	3.2	1.04	29	24	89	64	1.67
P5	2.8	3.0	0.46	70	21	168	141	n.a.
P6	2.0	3.5	1.12	32	24	54	81	1.28
P7	24.2	2.9	0.95	180	103	1307	725	1.85
P9	0.7	3.8	1.14	35	24	471	133	1.08

Pat., patient number; n.a., not available; AST, aspartate aminotransferase; ALT, alanine aminotransferase; GGT, gamma-glutamyl transferase; AP, alkaline phosphatase; INR, international normalized ratio.

Supplementary Table 2: SAXS parameters for the control and patient HSA samples

SAMPLE	Rg +/- (Å) from Guinier plot		Rg(Å) from P(r)	I(0) from Guinier plot	Kratky plot (Area)	D_{max} (Å) [a]	Simple SAXS [b] ID #	SASBDB [b] ID #
**HSA**	28.19	0.15	27.8	2765	2.9129	83	HHF44M	SASDLC4
**HMA**	28.52	0.12	28.03	2819	2.9608	86	HHF44M	SASDL24
**HNA1**	28.61	0.11	28.09	2788	2.9922	86	QE6ZEW	SASDL34
**HNA2**	28.16	0.10	27.75	2892	2.9135	82	D2QA9M	SASDL44
**C1**	27.47	0.19	27.73	63.7	2.7104	83	OVNNRM	SASDLU3
**C2**	27.71	0.32	27.77	37.2	2.7579	83	SOSO0C	SASDLV3
**C3**	27.64	0.2	28.03	50.2	2.7555	83	ZAZTGF	SASDLW3
**C4**	27.79	0.27	28.3	40.5	2.8067	83	FDDSDV	SASDLX3
**C6**	27.73	0.32	28.02	127.2	2.7628	83	0LTRHX	SASDLY3
**P1**	29.12	0.13	28.85	2924	3.0882	93	KWRALZ	SASDL54
**P2**	29.39	0.14	29.19	2816	3.1228	95	QGGZ1H	SASDL64
**P3**	28.69	0.11	28.37	2821	3.0177	90	CSCOGC	SASDL74
**P4**	28.56	0.28	28.86	44.1	3.0807	91	H9OQM3	SASDL84
**P5**	28.24	0.56	28.68	143.4	2.9394	89	D5550D	SASDL94
**P6**	28.17	0.22	28.29	170.6	2.8393	85	NHAPBC	SASDLA4
**P7**	28.04	0.4	28.57	136.5	2.8705	88	M4ZFDD	SASDLD4
**P9**	28.07	0.64	28.69	181.2	2.9433	89	WZAUQJ	SASDLE4

[a] Maximal dimension (Dmax) defined from pair distribution function (P(r)) calculated by SCATTER.

[b] SAXS data and models are deposited in Simple SAXS database (https://simplescattering.com/) and SASBDB (https://www.sasbdb.org/).

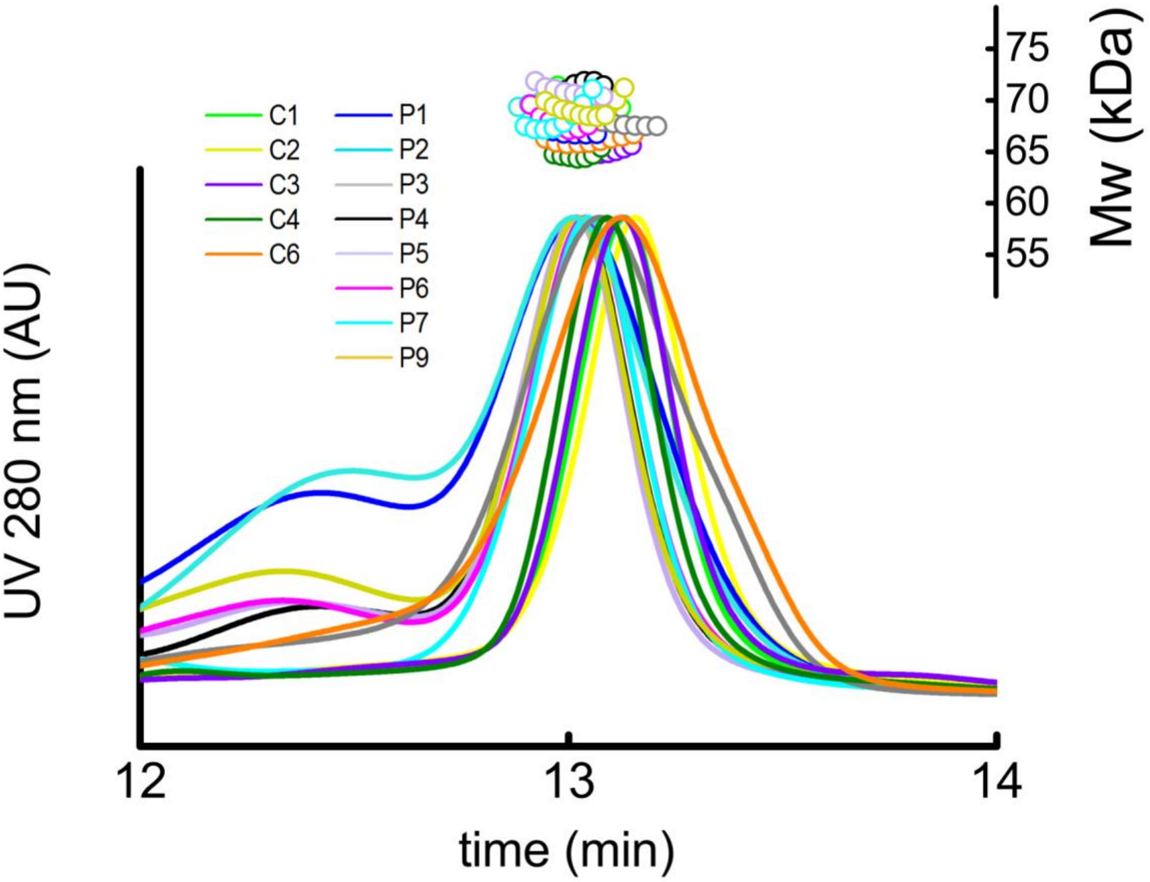

Supplementary Fig. 1 SEC-MALS chromatograms.

SEC-MALS chromatograms for the control and patient HSA samples. Solid lines represent the UV signal at 280 nm. In contrast, symbols represent molecular mass determined by MALS versus elution volume.

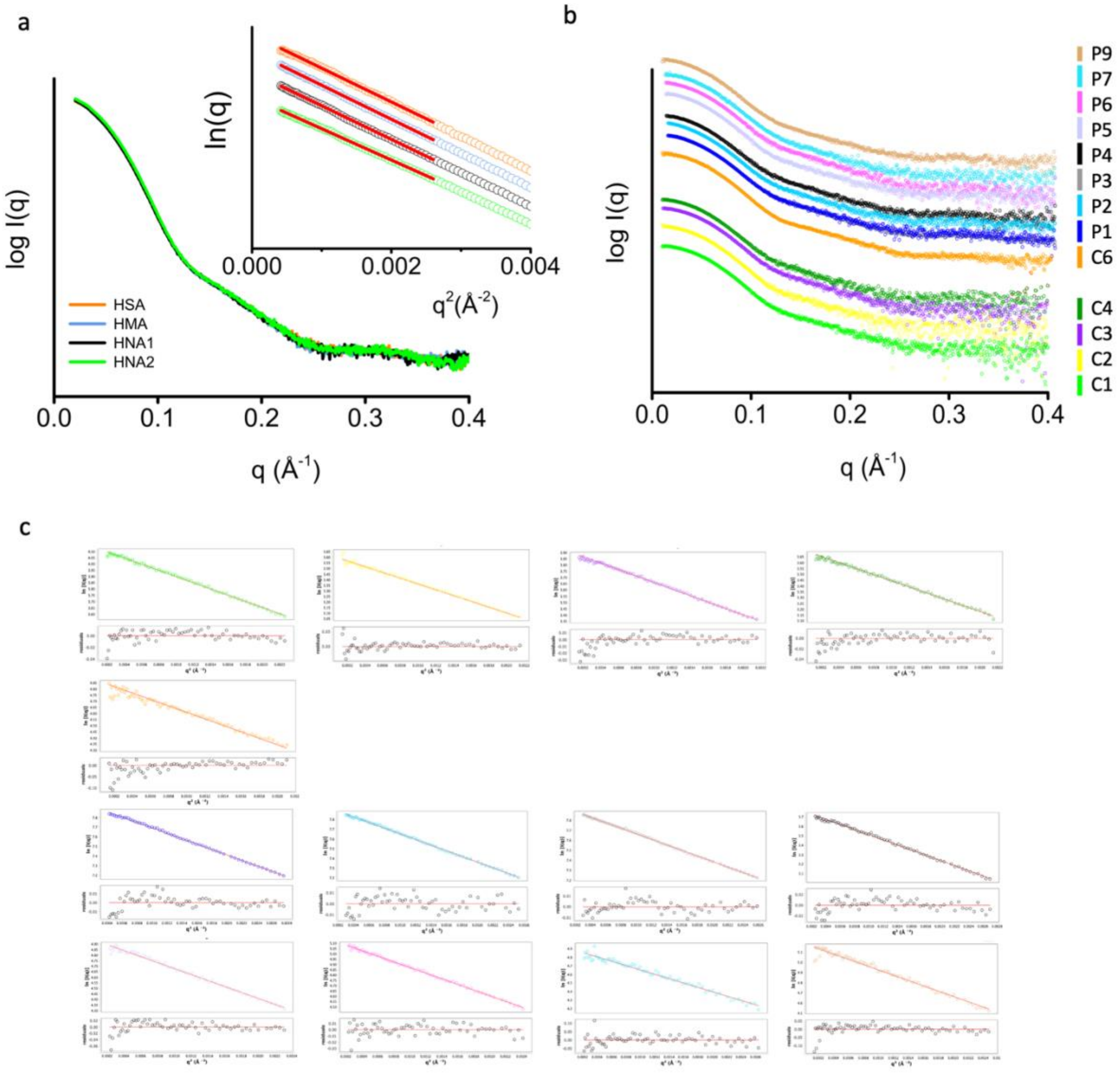

Supplementary Fig. 2 Experimental SAXS curves.

(a) Final merged SAXS curves for HMA, HNA1, HNA2, and HSA control sample. Inset: Corresponding Guinier plots with q*Rg <1.5 limit. Guinier plots were used to determine Rg values listed in Supplementary Table 2.

(b) Final merged SAXS curves for healthy controls' (C1 - C4), patients' (P1 - P7, P9), and commercial HSA (C6). (c) Corresponding Guinier plots with q*Rg <1.5 limit for all SAXS curves shown in panel b. The color code is the same as in panel b. Guinier plots were used to determine Rg values used in Fig. 1e and listed in Supplementary Table 2.
