# Supplementary material for: Albumin in patients with liver disease shows an altered conformation
Source: Commun Biol. 2021 Jun 14;4:731. doi: 10.1038/s42003-021-02269-w (PMC8203801; doi:10.1038/s42003-021-02269-w)
Supplement: Supplementary file 3 — Reporting Summary [file 42003_2021_2269_MOESM3_ESM.pdf]

nature research

Corresponding author(s): Michal Hammel and Karl Oettl

Last updated by author(s): 04-21-2021

Reporting Summary

Nature Research wishes to improve the reproducibility of the work that we publish. This form provides structure for consistency and transparency in reporting. For further information on Nature Research policies, see our Editorial Policies and the Editorial Policy Checklist.

Statistics

For all statistical analyses, confirm that the following items are present in the figure legend, table legend, main text, or Methods section.

n/a	Confirmed	
☐	☒	The exact sample size (*n*) for each experimental group/condition, given as a discrete number and unit of measurement
☐	☒	A statement on whether measurements were taken from distinct samples or whether the same sample was measured repeatedly
☐	☒	The statistical test(s) used AND whether they are one- or two-sided<br>*Only common tests should be described solely by name; describe more complex techniques in the Methods section.*
☒	☐	A description of all covariates tested
☒	☐	A description of any assumptions or corrections, such as tests of normality and adjustment for multiple comparisons
☒	☐	A full description of the statistical parameters including central tendency (e.g. means) or other basic estimates (e.g. regression coefficient) AND variation (e.g. standard deviation) or associated estimates of uncertainty (e.g. confidence intervals)
☒	☐	For null hypothesis testing, the test statistic (e.g. *F*, *t*, *r*) with confidence intervals, effect sizes, degrees of freedom and *P* value noted<br>*Give P values as exact values whenever suitable.*
☒	☐	For Bayesian analysis, information on the choice of priors and Markov chain Monte Carlo settings
☒	☐	For hierarchical and complex designs, identification of the appropriate level for tests and full reporting of outcomes
☐	☒	Estimates of effect sizes (e.g. Cohen's *d*, Pearson's *r*), indicating how they were calculated

Our web collection on statistics for biologists contains articles on many of the points above.

Software and code

Policy information about availability of computer code

Data collection	Beamline 12.3.1 (ALS) - Blu-Ice, Classen et al., 2013 (https://www.ncbi.nlm.nih.gov/pubmed/23396808);
Data analysis	SCÅTTER Developed by Dr. Robert Rambo at SIBYLS, now at the Diamond Light Source (Didcot, UK) (http://www.bioisis.net/tutorial/9); GNOM (Svergun, D. et al., 1992) (https://www.embl-hamburg.de/biosaxs/gnom.html); SREFLEX (Panjkovich et al. 2016) (https://www.embl-hamburg.de/biosaxs/sreflex.html); FoXS (Schneidman-Duhovny et al., 2013) (https://modbase.compbio.ucsf.edu/foxs/); Multi-FoXS (Schneidman-Duhovny et al., 2016) (https://modbase.comapbio.ucsf.edu/multifoxs/); CHIMERA (Pettersen et al., 2004) (https://www.cgl.ucsf.edu/chimera/); OriginLab 2016 Origin (OriginLab, Northampton, MA) (https://www.originlab.com/);

For manuscripts utilizing custom algorithms or software that are central to the research but not yet described in published literature, software must be made available to editors and reviewers. We strongly encourage code deposition in a community repository (e.g. GitHub). See the Nature Research guidelines for submitting code & software for further information.

Data

Policy information about availability of data

All manuscripts must include a data availability statement. This statement should provide the following information, where applicable:

- Accession codes, unique identifiers, or web links for publicly available datasets
- A list of figures that have associated raw data
- A description of any restrictions on data availability

SEC-SAXS-MALS data, including atomistic models, are deposited in the Simple SAXS data bank (https://simplescattering.com/) and SASBDB (https://www.sasbdb.org/). IDs are listed in Supplementary Table 2.

Field-specific reporting

Please select the one below that is the best fit for your research. If you are not sure, read the appropriate sections before making your selection.

- [x] Life sciences
- [ ] Behavioural & social sciences
- [ ] Ecological, evolutionary & environmental sciences

For a reference copy of the document with all sections, see nature.com/documents/nr-reporting-summary-flat.pdf

Life sciences study design

All studies must disclose on these points even when the disclosure is negative.

Sample size	No sample-size calculation was performed. The presented study represents a pilot study as there was no evidende of differences in HSA structure of patients and healthy donors. After measuring the herein presented samples it turned out that differences between the albumin samples can be detected.
Data exclusions	Due to technical problems it was not possible to measure a complete SEC-SAXS-MALS data set for HSA of patient 8 that is why we had to exclude it. We also measured recombinant albumin produced in rice (C5). We excluded this control as all other samples of the study were human samples.
Replication	SAXS experiments for control C6 (Sigma HSA) was repeated 3 times independently. HMA, HNA1, and HNA2 fraction, patients' and healthy donors HSA were collected one time. We confirm that all attempts to replicate experiments were successful.
Randomization	Participants with a diagnosis of decompensated cirrhosis were allocated to the patient group, whereas healthy volunteers (without any diagnosed liver disease) were allocated to the control group.
Blinding	Investigators were blinded to group allocation during SEC-SAXS-MALS Data collection and subsequent analyses.

Reporting for specific materials, systems and methods

We require information from authors about some types of materials, experimental systems and methods used in many studies. Here, indicate whether each material, system or method listed is relevant to your study. If you are not sure if a list item applies to your research, read the appropriate section before selecting a response.

Materials & experimental systems

n/a	Involved in the study	
x		Antibodies
x		Eukaryotic cell lines
x		Palaeontology and archaeology
x		Animals and other organisms
	x	Human research participants
	x	Clinical data
x		Dual use research of concern

Methods

n/a	Involved in the study	
x		ChIP-seq
x		Flow cytometry
x		MRI-based neuroimaging

Human research participants

Policy information about studies involving human research participants

Population characteristics	8 patients (2 females and 6 males) aged 42 – 81 years with decompensated cirrhosis and 4 healthy control persons (2 women and two 2 men) aged 23 – 55 years were included in the study.
Recruitment	Patients were recruited by the responsible investigator (medical doctor) at the hospital's GI ward. We included 8 patients with decompensated cirrhosis admitted to our GI ward for treatment of tense ascites refractory to diuretics. All patients underwent large-volume paracentesis followed by albumin infusion (10 g per liter ascites). Blood samples for albumin preparation were collected prior to paracentesis and subsequent albumin infusion, respectively. Controls were recruited within the Division of Physiological Chemistry, Otto-Loewi-Research Center, Medical University of Graz.<br>All patients and healthy control persons were informed both orally and in writing and gave their written informed consent before their inclusion in the study.
Ethics oversight	The study protocol was approved by the ethics committee of the Medical University of Graz (registered at the Office for Human Research Protections of the US Departments of Health and Human Services: IRB00002556). Votes: 29-040 ex 16/17 and 29-460 ex 16/17.

Note that full information on the approval of the study protocol must also be provided in the manuscript.

Clinical data

Policy information about clinical studies

All manuscripts should comply with the ICMJEguidelines for publication of clinical research and a completedCONSORT checklist must be included with all submissions.

Clinical trial registration	The respective study does not represent any clinical trial.
Study protocol	Not available, see above.
Data collection	Sample collection was conducted at the Department of Internal Medicine, Division of Gastroenterology and Hepatology, Medical University of Graz, between January 2017 and December 2018. Clinical laboratory values were analyzed by the clinical in-house laboratory. Preparation of HSA from plasma and therof characterization was performed at the Division of Physiological Chemistry, Otto-Loewi Research Center, Medical University of Graz.
Outcomes	Not available, as the respective study was no clinical trial.
